# P-1719. Promoting antibiotic stewardship for commercial poultry production in Bangladesh: using social and behavioral change communication approach

**DOI:** 10.1093/ofid/ofae631.1884

**Published:** 2025-01-29

**Authors:** S M Murshid Hasan

**Affiliations:** Mahidol University, Dhaka, Dhaka, Bangladesh

## Abstract

**Background:**

Bangladesh has a high prevalence of antimicrobial resistance due to extensive antibiotic use in humans and animals. This study aimed to develop social and behavioral change communication (SBCC) strategies to promote antibiotic stewardship in commercial poultry production in Bangladesh.

**Methods:**

In 2022, we used formative research findings to explore contextual factors of antibiotic sale, prescribing, promotion, and use among four groups in Narsingdi, Bangladesh: 1) poultry farmers, 2) pharmacy shop employees, 3) veterinarians, and 4) pharmaceutical representatives. We conducted multiple intervention design workshops to select target behaviors and develop SBCC messaging. We used the behavior change wheel model to analyze the data and summarize the findings.

**Results:**

Participants identified their behaviors considered to be changed and assisted in developing SBCC for all four stakeholder groups. Poultry farmers had two behaviors to change: not following infection control measures and irrational antibiotic use. SBCC target behaviors included taking appropriate infection control measures, seeking registered veterinarian consultations, and rational antibiotic use. Pharmacy shop employees had one behavior to change: unnecessary antibiotic dispensing practices. SBCC target behaviors included asking farmers for prescriptions and referring them to registered veterinarians. Registered veterinarians had one behavior to change: unnecessary antibiotic prescribing practices. SBCC target behaviors included appropriate antibiotic prescribing practices and increasing farmer awareness for rational antibiotic use. Pharmaceutical sales representatives had one behavior to change: unethical antibiotic promotion. SBCC target behaviors included appropriate antibiotic promotion.

**Conclusion:**

We identified stakeholders considered behavior change and SBCC intervention delivery to them to promote antibiotic stewardship for commercial poultry production, including improved health-seeking behaviors, and improved health literacy on antibiotic transactions. The study findings may contribute to developing health communication materials to promote antibiotic stewardship among stakeholders in commercial poultry production in Bangladesh.

**Disclosures:**

**All Authors**: No reported disclosures

